# Model correction of diagnostic coding-based RSV incidence for children 0–4 years in the US

**DOI:** 10.1186/s12879-024-09474-y

**Published:** 2024-06-21

**Authors:** Sabina O. Nduaguba, Phuong T. Tran, Almut G. Winterstein

**Affiliations:** 1 Department of Pharmaceutical Systems and Policy, College of Pharmacy, West Virginia University, Morgantown, WV USA; 2 West Virginia University Cancer Institute, Morgantown, WV USA; 3 Department of Pharmaceutical Outcomes and Policy, College of Pharmacy, University of Florida, 1225 Center Drive, PO Box 100496, Gainesville, Florida, FL 32611 USA; 4 Faculty of Pharmacy, HUTECH University, Ho Chi Minh City, Vietnam; 5 Center for Drug Evaluation and Safety, University of Florida, Gainesville, FL USA; 6 Department of Epidemiology, College of Medicine and College of Public Health and Health Professions, University of Florida, Gainesville, FL USA

**Keywords:** Respiratory syncytial virus, RSV epidemiology, National Respiratory and Enteric Virus Surveillance System, International classification of diseases, Healthcare data

## Abstract

**Background:**

Although administrative claims data have a high degree of completeness, not all medically attended Respiratory Syncytial Virus-associated lower respiratory tract infections (RSV-LRTIs) are tested or coded for their causative agent. We sought to determine the attribution of RSV to LRTI in claims data via modeling of temporal changes in LRTI rates against surveillance data.

**Methods:**

We estimated the weekly incidence of LRTI (inpatient, outpatient, and total) for children 0–4 years using 2011–2019 commercial insurance claims, stratified by HHS region, matched to the corresponding weekly NREVSS RSV and influenza positivity data for each region, and modelled against RSV, influenza positivity rates, and harmonic functions of time assuming negative binomial distribution. LRTI events attributable to RSV were estimated as predicted events from the full model minus predicted events with RSV positivity rate set to 0.

**Results:**

Approximately 42% of predicted RSV cases were coded in claims data. Across all regions, the percentage of LRTI attributable to RSV were 15–43%, 10–31%, and 10–31% of inpatient, outpatient, and combined settings, respectively. However, when compared to coded inpatient RSV-LRTI, 9 of 10 regions had improbable corrected inpatient LRTI estimates (predicted RSV/coded RSV ratio < 1). Sensitivity analysis based on separate models for PCR and antigen-based positivity showed similar results.

**Conclusions:**

Underestimation based on coding in claims data may be addressed by NREVSS-based adjustment of claims-based RSV incidence. However, where setting-specific positivity rates is unavailable, we recommend modeling across settings to mirror NREVSS’s positivity rates which are similarly aggregated, to avoid inaccurate adjustments.

**Supplementary Information:**

The online version contains supplementary material available at 10.1186/s12879-024-09474-y.

## Background

Respiratory syncytial virus (RSV) is a common causative pathogen for lower respiratory tract infection (LRTI) among infants and young children [1–4] and severe disease among older adults [5, 6]. RSV infection is seasonal and, in the US, the peak season occurs during the winter months between November and March [7]. Globally, 33.1 million episodes of RSV-associated LRTI occur annually among children < 5 years old, resulting in close to 3 200 000 hospital admissions and 56 600 deaths [8]. In the US, RSV-related outpatient visits range from 108 to 157/1 000 children-years among children < 6 months to 31–77/1 000 children years among children 2–4 years [9]. For more severe cases, RSV infection results in emergency department visits and hospitalization rates of 45–68 and 12–22/1 000 children-years, respectively, among children < 6 months to 11–15 and less than 1/1 000 children-years among children 2–5 years [9], with an estimated 50 000–60 000 hospitalizations in a season among children < 2 years old [10]. 

Epidemiologic estimates of the burden of RSV infections as described above are typically derived from prospective cohorts, which may be limited in sample size and generalizability, or from automated healthcare data collected during routine clinical care of cases. Administrative claims data are an important source of health services utilization data repurposed for this type of epidemiologic research. Although claims data offer the advantage of having a high degree of completeness due to their use for reimbursement, not all medically attended LRTI are tested for RSV and assigned respective International Classification of Disease (ICD) codes that facilitate measurement of RSV-specific LRTI incidence rates. This could potentially underestimate RSV incidence in claims data. To correct for underestimation, LRTI cases that might be attributable to RSV can be modelled against data with RSV positivity to determine the proportion of cases due to RSV. Two previous studies modelled LRTI hospitalizations collected in the Healthcare Cost and Utilization Project (HCUP) data against RSV test positivity data collected by National Respiratory and Enteric Virus Surveillance System (NREVSS) from the Centers for Disease Control and Prevention (CDC) [11, 12]. Importantly, because NREVSS does not provide information on patient characteristics (age, disease type or severity) or the setting (inpatient/outpatient), the models had to be based on the assumption that overall RSV positivity results provided by NREVSS are representative of the RSV positivity in the study population, i.e., LRTI hospitalizations. However, we have demonstrated previously that the propensity to test for RSV as well as RSV positivity varies appreciably by patient age, disease type and severity, and setting [13] and thus, use of aggregate RSV positivity information as available from NREVSS may yield inaccurate adjustments for RSV undertesting when analyzing claims data.

We aimed to estimate the proportion of LRTI episodes attributable to RSV and evaluate the performance of NREVSS-based RSV incidence correction models in claims data when applied separately to inpatient versus outpatient LRTI episodes.

## Methods

### Study design and population

We utilized a retrospective cohort study design to model 2011—2019 MarketScan® Commercial Claims and Medicare Supplemental data (MarketScan) and NREVSS virology testing data obtained from the U.S. Centers for Disease Control and Prevention (CDC) corresponding to the same time period. Participants were children in MarketScan 0 to 4 years of age who were continuously enrolled across an RSV year (July-June), in non-capitated health plans, and with prescription benefits. Enrollees in capitated health plans were excluded to avoid incomplete documentation of services received. Continuous enrollment was defined as a gap of less than 3 days between the end of one enrollment period and the next. The University of Florida Institutional Review Board exempt the study from review due to use of de-identified data.

### MarketScan® commercial claims and medicare supplemental data (MarketScan)

MarketScan provides administrative claims data for a national sample of persons across the US with employer-sponsored health insurance, their spouses, and dependents. Data provided include information on beneficiary demographic characteristics, medical encounters with associated diagnoses and procedures, and dispensed prescription drugs. Over 130 million lives were covered between 2011 and 2019.

### Virology data

Weekly data on RSV and influenza tests are collated by the CDC through the National Respiratory and Enteric Virus Surveillance System (NREVSS) from a sample of clinical and public health laboratories across the US. The RSV-related data provide the total number of RSV tests conducted each week and the number of positive RSV tests, stratified by test type (antigen, viral isolation/culture, or polymerase chain reaction (PCR)) and by US Department of Health and Human Services (HHS) regions. Similarly, the influenza-related data include the total number of influenza tests conducted each week in each HHS region and the number of positive influenza tests for influenza A and B. Starting in 2015, NREVSS began to report influenza data separately for clinical and public health laboratories. We excluded 2015–2019 influenza data from public health laboratories because the data are restricted to positive specimens used mainly for surveillance to identify circulating strains. In both the RSV and Influenza datasets, weekly data start on the first Saturday of each year.

### Attribution of LRTI to RSV

We determined weekly LRTI incidence rates from MarketScan data in the inpatient and outpatient setting, stratified by age (0, 1, 2, 3 and 4 years) and HHS region. LRTI-related events were identified using ICD version 9 or 10 clinical modification codes considering codes that do or do not identify a specific pathogen (eTable 1). LRTIs with any specific pathogen (RSV or not) were included because all could have been candidates for RSV testing and contribute to RSV positivity results in NREVSS. To ensure that infection was the reason for hospital admission, we included principal diagnoses of LRTI as well as secondary diagnoses of LRTI with a primary diagnosis of a medical condition that is directly related to the infection, such as respiratory distress (eTable 2). A unique RSV episode was defined as a cluster of LRTI-related claims 30 or more days apart from the next adjacent claim. Inpatient episodes were identified before outpatient episodes with ± 30 days around each inpatient episode excluded from the outpatient risk period. This hierarchical approach prioritizing inpatient episodes was to ensure that all severe cases were captured. Weekly LRTI incidence rates, following the NREVSS definition of a week, were estimated as the total weekly counts of unique LRTIs (events) divided by the total number of days at risk (population-time) in each week.

### Preparation of analytical dataset

We used corresponding weekly NREVSS data for RSV and influenza to determine the proportion of LRTI events attributable to RSV. RSV and influenza positivity rates were, respectively, determined as proportion of total number of RSV and influenza tests conducted that week that were positive. We then modeled the weekly number of LRTI events against RSV and influenza positivity rates (NREVSS data) using negative binomial models with days at risk as the log offset variable as shown in Eq. 1.

1 $$\eqalign{{{\rm{Y}}_{{\rm{(i)}}}}{\rm{ = }} & {\rm{\alpha exp (}}{{\rm{\beta }}_{\rm{0}}}{\rm{ + }}{{\rm{\beta }}_{{\rm{1()}}}}\left[ {{{\rm{t}}_{\rm{i}}}} \right]{\rm{ + }}{{\rm{\beta }}_{\rm{2}}}\left[ {{{\rm{t}}_{\rm{i}}}^{\rm{2}}} \right]{\rm{ + }}{{\rm{\beta }}_{\rm{3}}}\left[ {{{\rm{t}}_{\rm{i}}}^{\rm{3}}} \right]{\rm{ + }}{{\rm{\beta }}_{\rm{4}}}\left[ {{{\rm{t}}_{\rm{i}}}^{\rm{4}}} \right]{\rm{ }}\,{\rm{ + }} \cr & {{\rm{\beta }}_{\rm{5}}}\left[ {{\rm{sin }}\left( {{\rm{2}}{{\rm{t}}_{\rm{i}}}{\rm{\pi /52}}{\rm{.15}}} \right)} \right]{\rm{ + }}{{\rm{\beta }}_{\rm{6}}}{\rm{[cos (2}}{{\rm{t}}_{\rm{i}}}{\rm{\pi /52}}{\rm{.15)] + }} \cr & {{\rm{\beta }}_{\rm{7}}}\left[ {{\rm{Influenza \,A}}} \right]{\rm{ + }}{{\rm{\beta }}_{\rm{8}}}\left[ {{\rm{Influenza \,B}}} \right]{\rm{ + }}{{\rm{\beta }}_{\rm{9}}}\left[ {{\rm{RSV}}} \right]{\rm{)}} \cr}$$

where Y _(i)_ represents the number of LRTI events during a given week i, α is the offset term and is equal to the log of the population size in each age group and region, t is a running index of the weeks between July 2011 to June 2019 where 1 is assigned to week 1, 2 to week 2 and so on, sin and cos are harmonic functions of t accounting for seasonal events, and β7 through β9 represent coefficients associated with the proportion of standardized specimens testing positive during a given week in the NREVSS data [12, 14]. Age was not an input parameter for the model as separate predictions were made for each age stratum. Because the majority of RSV positivity results are based on either antigen or PCR results [13], we considered positivity results from both test types in our model. The RSV variable was a combination of antigen and PCR positivity rates weighted by the weekly distribution of tests conducted. For example, if the total number of antigen and PCR tests conducted in a week were 200 and 300 respectively, the positivity rates for the corresponding tests in NREVSS data for that week were weighted by 0.4 and 0.6 respectively and then summed to obtain the weighted RSV positivity rates. This assumed that the distribution of tests by test type was similar in both the NREVSS and MarketScan cohort from which data was collected.

### Analysis

To estimate the number of LRTI events attributable to RSV, we adapted the approach by Zhou et al. [11] We estimated the predicted number of events with all parameters defined in the full model and with RSV positivity rate set to 0 (reduced model). The predicted number of events in the reduced model was then subtracted from the predicted number of events in the full model to obtain the number of LRTI events attributable to RSV. We used the 95% confidence interval for the RSV coefficient in the reduced model to calculate 95% upper and lower confidence limits for the number of RSV cases attributed to LRTI. We deviated from Zhou et al.’s approach by not excluding LRTI cases with diagnostic codes specifying RSV or influenza from the model. In their approach, only LRTIs without RSV or influenza designation were modeled and the estimated excess RSV cases were then added to the excluded RSV cases in the claims data. This approach could potentially mis-specify the model and result in overestimation of RSV incidence rates, because NREVSS positivity data is a reflection of tests ordered for all LRTI cases (i.e., with or without confirmed RSV).

Separate models were run by HHS region and then aggregated to obtain the total number of LRTI events attributed to RSV. Strata with negative estimates were substituted with 0. RSV incidence was estimated as the number of predicted RSV cases attributed to LRTI divided by the total number of person-years at risk for LRTI. We conducted sensitivity analyses considering RSV test types. Specifically, to check the robustness of our modeling approach and assumptions, separate models estimating the attribution of RSV to LRTI were run for antigen and PCR tests. This had the same assumption as the initial model but had the potential to overestimate RSV-attributable LRTI episodes since the predictor was all LRTI episodes identified in MarketScan data. Hence, the weekly attribution from each model was weighted by the weekly distribution of tests before aggregation.

All analyses were conducted using SAS 9.04.01.M6.

## Results

Across July 2011 to June 2019, there were 54 872 and 1 432 300 unique episodes coded for LRTI in the inpatient and outpatient files in MarketScan for a total of 1 487 172, making up 42.1% of the 351 258 model-predicted RSV-attributable LRTI episodes (Table 1). Nationally, the percentage of LRTI episodes coded as involving RSV pathogen was 40.0% (range: 35.1% (Boston) − 44.3% (Seattle)) in the inpatient setting with smaller percentages in the outpatient setting [8.8% (range: 5.8% (San Francisco) − 12.0% (Dallas)]. Based on model predictions, we estimate that, nationally, 29% (range: 15% (Philadelphia) -43% (Atlanta)) and 24% (range: 10% (San Francisco) -31% (Atlanta)) of inpatient and outpatient LRTI episodes are, respectively, attributable to RSV. For the two settings combined, the percentage of LRTI episdoes coded as involving RSV pathogen was 10.0% (range: 6.9% (San Francisco) -12.9% (Dallas)) with 24% (10% (San Francisco) -31% (Atlanta)) of LRTI episodes were attributable to RSV.

**Table 1:** Number and Percentages of LRTI episodes Coded as RSV and attributed to RSV by Model Estimation for children 0–4 Years in the US

Region	LRTI Episodes (*N*)	LRTI Episodes coded as RSV *N* (%)	Model-Based RSV-Attributable LRTI Episodes *N* (95% CI)	Proportion of LRTIs attributed to RSV (95% CI)
**Inpatient**				
Boston	1,976	693 (35.1)	560 (288–785)	0.28 (0.15–0.40)
New York	5,547	2,286 (41.2)	942 (341-1,470)	0.17 (0.06–0.26)
Philadelphia	4,478	1,804 (40.3)	693 (141-1,170)	0.15 (0.03–0.26)
Atlanta	10,801	4,335 (40.1)	4,630 (3801-5,356)	0.43 (0.35–0.49)
Chicago	10,091	3,909 (38.7)	2,640 (1,664-3,497)	0.26 (0.16–0.34)
Dallas	8,833	3,677 (41.6)	3,254 (2,492-3,915)	0.37 (0.28–0.44)
Kansas	3,275	1,372 (41. 9)	972 (506-1,354)	0.30 (0.15–0.41)
Denver	3,309	1,357 (41.0)	1,114 (711-1,451)	0.34 (0.21–0.44)
San Francisco	4,837	1,772 (36.6)	767 (164-1,288)	0.16 (0.03–0.27)
Seattle	1,725	764 (44.3)	511 (210–749)	0.30 (0.12–0.43)
**Total**	54,872	21,969 (40.0)	16,082 (10,318 − 21,035)	0.29 (0.19–0.38)
**Outpatient**				
Boston	42,732	3,027 (7.1)	8,920 (6,501 − 11,165)	0.21 (0.15–0.26)
New York	147,096	10,170 (6.9)	23,200 (15,073 − 30,788)	0.16 (0.10–0.21)
Philadelphia	99,313	8,057 (8.1)	17,145 (11,596 − 22,312)	0.17 (0.12–0.22)
Atlanta	351,159	35,483 (10.1)	106,807 (91,896 − 120,790)	0.31 (0.26–0.34)
Chicago	241,333	16,124 (6.7)	54,513 (41,438 − 66,620)	0.23 (0.17–0.28)
Dallas	278,293	33,310 (12.0)	79,578 (65,987 − 92,145)	0.29 (0.24–0.34)
Kansas	67,059	6,432 (9.6)	14,276 (9,607 − 18,511)	0.21 (0.14–0.28)
Denver	37,103	3,740 (10.1)	10,917 (8,540 − 13,073)	0.30 (0.23–0.36)
San Francisco	126,887	7,299 (5.8)	12,683 (3,828 − 20,836)	0.10 (0.03–0.17)
Seattle	41,325	2,382 (5.8)	10,740 (8,349 − 12,939)	0.26 (0.20–0.31)
**Total**	1,432,300	126,024 (8.8)	338,779 (262,816 − 409,179)	0.24 (0.18–0.29)
**Combined**				
Boston	44,708	3,720 (8.3)	9,448 (6,969 − 11,751)	0.21 (0.15–0.26)
New York	152,643	12,456 (8.2)	23,848 (15,530 − 31,623)	0.16 (0.10–0.21)
Philadelphia	103,791	9,861 (9.5)	17,623 (11,914 − 22,946)	0.17 (0.11–0.22)
Atlanta	361,960	39,818 (11.0)	110,531 (95,431 − 124,705)	0.31 (0.26–0.35)
Chicago	251,424	20,033 (8.0)	56,478 (43,091 − 68,893)	0.23 (0.17–0.28)
Dallas	287,126	36,987 (12.9)	81,799 (68,088–94,503)	0.29 (0.24–0.33)
Kansas	70,334	7,804 (11.1)	15,081 (10,248 − 19,470)	0.22 (0.15–0.28)
Denver	40,412	5,097 (12.6)	12,000 (9,503 − 14,273)	0.30 (0.24–0.36)
San Francisco	131,724	9,071 (6. 9)	13,248 (4,280 − 21,521)	0.10 (0.03–0.16)
Seattle	43,050	3,146 (7.3)	11,202 (8,766 − 13,445)	0.26 (0.20–0.31)
**Total**	1,487,172	147,993 (10.0)	351,258 (273,820 − 423,130)	0.24 (0.19–0.29)

**Fig. 1:**
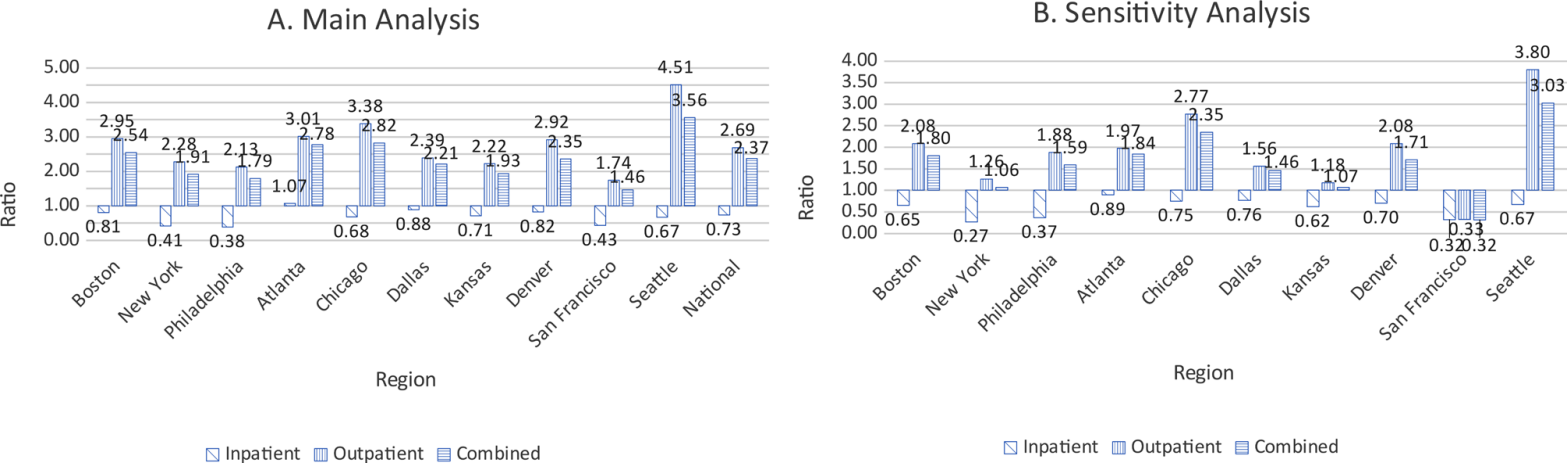
Ratio of number of RSV-coded LRTI episodes to model-estimated number of RSV-LRTI episodes for children 0–4 years in the US

Table 2 shows estimated RSV LRTI incidence rates based on the modelling of LRTI episodes from MarketScan data against RSV and influenza testing and positivity from NREVSS data. The overall inpatient RSV LRTI incidence was 1.74 (1.11–2.27) cases per 1 000 person-years, ranging between 0.85 (0.18–1.43, San Francisco) and 3.04 (1.94–3.96, Denver) cases per 1 000 person-years. The outpatient incidence was 38.55 (29.91–46.56, ranging between 14.75 (4.45–24.23, San Francisco) and 60.85 (50.46–70.46, Dallas). Figure 1 shows the ratio of number of model-estimated RSV LRTI (LRTI episodes attributed to RSV) to number of LRTI cases coded for RSV in MarketScan data (LRTI episodes with an RSV diagnostic code). Nationally, the ratio of number of model-estimated RSV LRTI to RSV-coded LRTI episodes was 0.73, 2.69, and 2.37 for inpatient and outpatient cases and combined, respectively, i.e., the model predicted fewer inpatient RSV-LRTI episodes than were coded as RSV-associated. for inpatient episodes. By region, this ranged from 0.38 to 1.07 for inpatient cases, 1.74–4.51 for outpatient cases, and 1.46–3.56 for both settings combined. With the exception of Atlanta, the ratio of predicted RSV to coded RSV cases was less than 1 in the inpatient setting while all regions had ratios greater than 1 for the outpatient setting and both settings combined. Sensitivity analyses based on separate models for the RSV test types showed similar estimates of ratios less than 1 for inpatient ), and greater than 1 for outpatient and combined settngs.

**Table 2:** Model-estimated annual RSV incidence for children 0–4 years in the US

Region	Inpatient (Cases/100,000 PY)	Outpatient (Cases/100,000 PY)	Combined (Cases/100,000 PY)
Boston	156(80–219)	2,590 (1,888-3,242)	1,343 (991-1,670)
New York	107 (39–167)	2,790 (1,812-3,702)	1,393 (907-1,847)
Philadelphia	96 (20–162)	2,493 (1,686-3,244)	1,250 (845-1,627)
Atlanta	236 (194–273)	5,786 (4,978-6,543)	2,901 (2,505-3,274)
Chicago	147 (93–194)	3,173 (2,412-3,878)	1,606 (1,225-1,959)
Dallas	233 (179–281)	6,085 (5,046 − 7,046)	3,028 (2,520-3,498)
Kansas	218 (113–303)	3,366 (2,265-4,364)	1,732 (1,177-2,236)
Denver	304 (194–396)	3,086 (2,414-3,696)	1,667 (1,320-1,983)
San Francisco	85 (18–143)	1,475 (445-2,423)	752 (243-1,222)
Seattle	119 (49–175)	2,589 (2,012 − 3,119)	1,328 (1,039 − 1,594)
Total	174 (111–227)	3,855 (2,991-4,656)	1,946 (1,517-2,344)

*Abbreviations* LRTI = Lower Respiratory Tract Infection, PY = Person-Years; RSV = Respiratory Syncytial Virus

## Discussion

In this study, we applied modeling techniques to correct for underestimation of RSV-LRTI incidence rates common with administrative claims data. Taking advantage of seasonal variation of RSV and influenza infections, the model estimates temporal changes in LRTI incidences as a function of temporal changes in RSV and influenza test positivity data in NREVSS. The degree to which temporal changes in LRTI rates are explained by variations in positivity allows for the estimation of RSV attribution to LRTIs.

Our study findings arrive at similar estimate as a prospective study in 2009 which included 5 067 children < 5 years of age with acute respiratory illness (ARIs). The authors found that 20% of inpatient admissions, 18% of emergency visits and 15% of outpatient visits for ARI were attributable to RSV [9]. Although these numbers were lower than our model (29% for inpatients and 24% for outpatients), both studies indicate a larger proportion of inpatient visits were attributable to RSV when compared to outpatient visits. The RSV incidence per 1 000 children of this prospective study was 2.1–3.7 for hospitalizations and 61–80 for outpatient visits compared to our 1.7 and 38.5, respectively.

Our inpatient incidence of 173.67 per 100 000 children years is within the 47–360 range obtained for children 0–4 years using a similar modeling approach as ours applied to HCUP data [15]. Our estimate is, however, lower than the 514 per 100 000 children years obtained in a different modelling study which included a broad selection of medical conditions ranging from diseases and symptoms of the respiratory system to viral infections of unspecified site in National Inpatient Sample data [12], unlike our study which modeled only LRTIs. This difference in modeled disease between both studies may be more significant than other factors such as time period, cohort selection and data source, or modeling parameters.

Novel to our work in comparison to previous reports is the contrast of modeled RSV LRTI incidence estimates to those directly obtained from RSV-coded encounters in the claims data. The benchmark study by Zhou et al. reported that the model-based RSV hospitalization rate was about 74–76% that of ICD code-based rates for children under 5 years, i.e. a predicted to coded RSV ratio of 0.74–0.76 [11]. While this finding is probable, we argue that the conceptual underpinnings of the methodological approach used by the authors has a tendency to overestimate coded RSV. The authors excluded LRTI cases with RSV codes from their model and added the newly predicted cases from the model to those coded cases, In contrast, we considered *all* LRTI cases regardless of RSV coding to reflect the entirety of cases that would have contributed to the NREVSS data, expecting that the model corrects for RSV undercoding. Our approach resulted in model-predicted to coded RSV ratios greater than 1 for outpatient LRTIs but underestimated the contribution of RSV to inpatient LRTIs when compared to the coded data in MarketScan. In other words, correction for non-coding of RSV-associated inpatient LRTI events resulted in improbable estimates in 9 of the 10 HHS regions (Fig. 1). This finding is contrary to expectations since coding of RSV-LRTI in claims data is expected to undercount true RSV-attributable LRTI episodes, given that not all LRTI cases are tested for confirmatory pathogenic causation. Sensitivity analysis based on separate models for PCR and antigen-based positivity showed similar results.

A potential explanation for the unexpected finding is the lack of setting-specific positivity rates in NREVSS data. Given that the model for both inpatient and outpatient RSV-attributable LRTI are based on total RSV tests, it is conceivable that modeling inpatient LRTI rates from MarketScan data against RSV positivity rate based on total RSV tests from NREVSS data would result in inaccurate predictions which would minimally impart outpatient estimations since the majority of identified LRTI cases occur in the outpatient setting (1,432,300 vs. 54,872). This is significant as it highlights an important limitation of using NREVSS data to estimate RSV-LRTI [11]. We, therefore, recommend that in- and outpatient LRTIs are only modeled combined to align with RSV data from NREVSS and avoid inaccurate adjustments. Ideally, positivity rates used for modeling would be available for the target population that provides the cases for modeling, e.g., for the study at hand, positivity rates specific to children under age 5 years with LRTI stratified by setting would be used.

One of the strengths of our study is that it includes both inpatient and outpatient models unlike previous studies that only included an inpatient model, and that it allows a contrast between RSV attribution based on modeling versus ICD coding. It also addresses methodological limitations of those studies, such as the inclusion of all age groups and the assumed model distribution. It should be noted that even our combined estimates (across in- and outpatient episodes) or outpatient-specific estimates may be slightly inaccurate because NREVSS data are not specific to age or indication, even though most tests are expected to represent children of younger age.

A limitation of our study is that MarketScan data captures medically attended pediatric LRTIs only in children who are privately insured, resulting in underestimates of the overall disease burden in the US since RSV infections are less common among the commercially-insured compared to the publicly-insured population [16, 17]. Furthermore, a single LRTI episode could be associated with multiple encounters in both inpatient and outpatient files, which could potentially lead to double counting LRTI events. We minimized this limitation by applying an algorithm to identify unique LRTI inpatient and outpatient episodes requiring at least 30 days between individual episodes and applying a hierarchical algortihm that prioritized inpatient events. We were unable to model RSV-LRTI incidence by age because NREVSS data does not provide RSV positivity rate by age. Although most RSV cases occur in children under 5 years, the incidence of RSV is markedly different for each year of age from 0 to 4 years. Our model of LRTI incidence against RSV positivity aggregated across age takes into account this limitation. As discussed in our recent work, age-specific modeling by test type, test indication, clinical setting, and region should be a preferred method to better estimate RSV attribution to LRTIs [13]. 

## Conclusions

Underestimation based on coding in claims data may be addressed by NREVSS-based adjustment of claims-based RSV incidence. However, correction for under-coding of RSV-associated inpatient LRTI episodes resulted in improbable estimates of inpatient RSV LRTI incidence rates in most HHS regions. Where setting-specific positivity rates is unavailable, we recommend modelling claims data aggregated across settings, in order to NREVSS’s positivity rates which are similarly aggregated. This approach would avoid inaccurate adjustments.

### Electronic supplementary material

Below is the link to the electronic supplementary material.

Supplementary Material 1

Supplementary Material 2

## Data Availability

The data that support the findings of this study are available from Merative™ MarketScan® Research Databases (marketscan.support@merative.com) but restrictions apply to the availability of these data, which were used under license for the current study, and so are not publicly available.

## Abbreviations

ARI: Acute Respiratory Illness
CDC: Centers for Disease Control and Prevention
HCUP: Healthcare Cost and Utilization Project
HHS: United States Department of Health and Human Services
ICD: International Classification of Diseases
LRTI: Lower Respiratory Tract Infection
NREVSS: National Respiratory and Enteric Virus Surveillance System
PCR: Polymerase Chain Reaction
RSV: Respiratory Syncytial Virus
US: United States
