# Supplementary material for: Model correction of diagnostic coding-based RSV incidence for children 0–4 years in the US
Source: BMC Infect Dis. 2024 Jun 21;24:617. doi: 10.1186/s12879-024-09474-y (PMC11191139; doi:10.1186/s12879-024-09474-y)
Supplement: Supplementary file 1 — Supplementary Material 1 [file 12879_2024_9474_MOESM1_ESM.docx]

**eTable 1: International Classification of Diseases (ICD) Codes for Lower Respiratory Tract Infection**

| **ICD-9 Codes** | **Condition** | **IP** | **IP** | **OT** |
| --- | --- | --- | --- | --- |
|  |  | **1°^a^** | **2°** | **Any position** |
| 466 | Acute bronchitis and bronchiolitis | V | W | X |
| 466 | Acute bronchitis | V | W | X |
| 466.1 | Acute bronchiolitis | V | W | X |
| 466.11 | Acute bronchiolitis due to respiratory syncytial virus (RSV) | V | W | X |
| 466.19 | Acute bronchiolitis due to other infectious organisms | V | W | X |
| 480 | Viral pneumonia | V | W | X |
| 480.1 | Pneumonia due to respiratory syncytial virus | V | W | X |
| 480.2 | Pneumonia due to parainfluenza virus | V | W | X |
| 480.3 | Pneumonia due to SARS-associated coronavirus | V | W | X |
| 480.8 | Pneumonia due to other virus not elsewhere classified | V | W | X |
| 480.9 | Viral pneumonia, unspecified | V | W | X |
| 481 | Pneumococcal pneumonia | V | W | X |
| 482* | Other bacterial pneumonia | V | W | X |
| 483 | Pneumonia due to other specified organism | V | W | X |
| 483 | Pneumonia due to mycoplasma pneumoniae | V | W | X |
| 483.1 | Pneumonia due to chlamydia | V | W | X |
| 483.8 | Pneumonia due to other specified organism | V | W | X |
| 485 | Bronchopneumonia, organism unspecified | V | W | X |
| 486 | Pneumonia, organism unspecified | V | W | X |
| 490 | Bronchitis, not specified as acute or chronic | V | W | X |
| 487 | Influenza with pneumonia | V | W | X |
| 3.22 | Salmonella pneumonia | V | W | X |
| 011.6* | Tuberculosis pneumonia (any form) | V | W | X |
| 41.3 | Klebsiella pneumoniae | V | W | X |
| 73 | Ornithosis with pneumonia | V | W | X |
| 488.81 | Influenza due to identified novel influenza A virus with pneumonia | V | W | X |
| 488.11 | Influenza due to identified 2009 H1N1 influenza virus with pneumonia | V | W | X |
| 488.01 | Influenza due to identified avian influenza virus with pneumonia | V | W | X |
|  |  |  |  |  |
| **ICD-10 Codes** |  |  |  |  |
| J12* | Viral pneumonia, not elsewhere classified | V | W | X |
| J13* | Pneumonia due to Streptococcus pneumonia | V | W | X |
| J14* | Pneumonia due to Hemophilus influenza | V | W | X |
| J15*I | Bacterial pneumonia, not elsewhere classified | V | W | X |
| J16* | Pneumonia due to other infectious organisms, not elsewhere classified | V | W | X |
| J18* | Pneumonia, unspecified organism | V | W | X |
| J20* | Acute bronchitis | V | W | X |
| J21* | Acute bronchiolitis | V | W | X |
| J22* | Unspecified acute lower respiratory tract infection | V | W | X |
| J40 | Bronchitis, not specified as acute or chronic | V | W | X |
| A01.03 | Typhoid pneumonia | V | W | X |
| A02.22 | Salmonella pneumonia | V | W | X |
| A37.01 | Whooping cough due to Bordetella pertussis with pneumonia | V | W | X |
| A37.11 | Whooping cough due to Bordetella parapertussis with pneumonia | V | W | X |
| A37.81 | Whooping cough due to other Bordetella species with pneumonia | V | W | X |
| A37.91 | Whooping cough, unspecified species with pneumonia | V | W | X |
| A54.84 | Gonococcal pneumonia | V | W | X |
| B01.2 | Varicella pneumonia | V | W | X |
| B06.81 | Rubella pneumonia | V | W | X |
| B77.81 | Ascariasis pneumonia | V | W | X |
| B95.3 | Streptococcus pneumoniae as the cause of diseases classified elsewhere | V | W | X |
| B96.0 | Mycoplasma pneumoniae M. pneumoniae as the cause of diseases classified elsewhere | V | W | X |
| J09.X1 | Influenza due to identified novel influenza A virus with pneumonia | V | W | X |
| J10.0* | Influenza due to other identified influenza virus with pneumonia | V | W | X |
| J11.0* | Influenza due to unidentified influenza virus with pneumonia | V | W | X |

1°=Primary Diagnosis; 2°=Secondary Diagnosis; V=complementary primary diagnosis related to lower respiratory tract infection (LRTI); W= RSV LRTI; X=a stand-alone diagnosis
^a^Primary diagnosis to be combined with LRTI diagnosis in secondary position. See Table S4 for list of complementary primary diagnoses
