# Supplementary material for: Model correction of diagnostic coding-based RSV incidence for children 0–4 years in the US
Source: BMC Infect Dis. 2024 Jun 21;24:617. doi: 10.1186/s12879-024-09474-y (PMC11191139; doi:10.1186/s12879-024-09474-y)
Supplement: Supplementary file 2 — Supplementary Material 2 [file 12879_2024_9474_MOESM2_ESM.docx]

**eTable 2: Complementary Primary Diagnosis for Inpatient Lower Respiratory Tract Infection in Secondary Position**

| **ICD-9 Codes** | **Condition** |
| --- | --- |
| 310 | Pulmonary diseases due to other mycobacteria |
| 380 | Streptococcal septicemia |
| 382 | Pneumococcal septicemia |
| 383 | Septicemia due to anaerobes |
| 415 | Hemophilus influenzae (H. influenzae) infection in conditions classified elsewhere and of unspecified site |
| 488 | Influenza due to identified avian influenza virus |
| 490 | Bronchitis, not specified as acute or chronic |
| 769 | Respiratory distress syndrome in newborn |
| 793 | Rhinovirus infection in conditions classified elsewhere and of unspecified site |
| 796 | Respiratory syncytial virus (RSV) |
| 1124 | Candidiasis of lung |
| 1125 | Disseminated candidiasis |
| 1190 | Unspecified pulmonary tuberculosis confirmation unspecified |
| 1951 | Malignant neoplasm of thorax |
| 3812 | Methicillin resistant staphylococcus aureus septicemia |
| 3819 | Other staphylococcal septicemia |
| 3841 | Septicemia due to Hemophilus Influenzae (H. influenzae) |
| 3844 | Septicemia due to serratia |
| 3849 | Other septicemia due to gram-negative organisms |
| 4160 | Primary pulmonary hypertension |
| 4168 | Other chronic pulmonary heart diseases |
| 4185 | Other specified bacterial infections in conditions classified elsewhere and of unspecified site other gram-negative organisms |
| 4254 | Other primary cardiomyopathies |
| 4289 | Heart failure unspecified |
| 4290 | Myocarditis unspecified |
| 4871 | Influenza with other respiratory manifestations |
| 4878 | Influenza with other manifestations |
| 5060 | Bronchitis and pneumonitis due to fumes and vapors |
| 5078 | Pneumonitis due to other solids and liquids |
| 5100 | Empyema with fistula |
| 5109 | Empyema without fistula |
| 5119 | Unspecified pleural effusion |
| 5121 | Iatrogenic pneumothorax |
| 5130 | Abscess of lung |
| 5180 | Pulmonary collapse |
| 5181 | Interstitial emphysema |
| 5183 | Pulmonary eosinophilia |
| 5184 | Acute edema of lung unspecified |
| 5198 | Other diseases of respiratory system not elsewhere classified |
| 5199 | Unspecified disease of respiratory system |
| 7467 | Hypoplastic left heart syndrome |
| 7485 | Congenital agenesis hypoplasia and dysplasia of lung |
| 7702 | Interstitial emphysema and related conditions of newborn |
| 7707 | Chronic respiratory disease arising in the perinatal period |
| 7862 | Cough |
| 7866 | Swelling mass or lump in chest |
| 7907 | Bacteremia |
| 7991 | Respiratory arrest |
| 20013 | Lymphosarcoma involving intra-abdominal lymph nodes |
| 20020 | Burkitt's tumor or lymphoma unspecified site |
| 20290 | Other and unspecified malignant neoplasms of lymphoid and histiocytic tissue unspecified site |
| 27700 | Cystic fibrosis without meconium ileus |
| 27787 | Disorders of mitochondrial metabolism |
| 27904 | Congenital hypogammaglobulinemia |
| 27911 | Digeorge's syndrome |
| 28800 | Neutropenia, unspecified |
| 28804 | Neutropenia due to infection |
| 42292 | Septic myocarditis |
| 42299 | Other acute myocarditis |
| 42821 | Acute systolic heart failure |
| 42830 | Unspecified diastolic heart failure |
| 42832 | Chronic diastolic heart failure |
| 42840 | Unspecified combined systolic and diastolic heart failure |
| 42843 | Acute on chronic combined systolic and diastolic heart failure |
| 48802 | Influenza due to identified avian influenza virus with other respiratory manifestations |
| 48812 | Influenza due to identified 2009 H1N1 influenza virus with other respiratory manifestations |
| 48882 | Influenza due to identified novel influenza A virus with other respiratory manifestations |
| 48889 | Influenza due to identified novel influenza A virus with other manifestations |
| 49300 | Extrinsic asthma unspecified |
| 49301 | Extrinsic asthma with status asthmaticus |
| 49302 | Extrinsic asthma with (acute) exacerbation |
| 49310 | Intrinsic asthma unspecified |
| 49311 | Intrinsic asthma with status asthmaticus |
| 49312 | Intrinsic asthma with (acute) exacerbation |
| 49322 | Chronic obstructive asthma with (acute) exacerbation |
| 49390 | Asthma unspecified |
| 49391 | Asthma unspecified type with status asthmaticus |
| 49392 | Asthma unspecified with (acute) exacerbation |
| 51181 | Malignant pleural effusion |
| 51663 | Surfactant mutations of the lung |
| 51881 | Acute respiratory failure |
| 51882 | Other pulmonary insufficiency not elsewhere classified |
| 51883 | Chronic respiratory failure |
| 51884 | Acute and chronic respiratory failure |
| 51889 | Other diseases of lung not elsewhere classified |
| 51911 | Acute bronchospasm |
| 76502 | Disorders relating to extreme immaturity of infant 500-749 grams |
| 76503 | Disorders relating to extreme immaturity of infant 750-999 grams |
| 76515 | Disorders relating to other preterm infants 1250-1499 grams |
| 76516 | Disorders relating to other preterm infants 1500-1749 grams |
| 76517 | Disorders relating to other preterm infants 1750-1999 grams |
| 76523 | 25-26 completed weeks of gestation |
| 77012 | Meconium aspiration with respiratory symptoms |
| 77018 | Other fetal and newborn aspiration with respiratory symptoms |
| 77084 | Respiratory failure of newborn |
| 77088 | Hypoxemia of newborn |
| 77089 | Other respiratory problems after birth |
| 77181 | Septicemia [sepsis] of newborn |
| 77984 | Meconium staining |
| 78607 | Wheezing |
| 78609 | Other respiratory abnormalities |
| 79901 | Asphyxia |
| 79902 | Hypoxemia |
| 96502 | Poisoning by methadone |
| 99591 | Sepsis |
| 99592 | Severe sepsis |
| 99609 | Other mechanical complication of cardiac device implant and graft |
| 99739 | Other respiratory complications |
| 0320, 0321, 0322, 0323, 0340, 460-465, 473, 474, 4781, 78491, 3801-3802, 3810-3814, 382 | Upper respiratory tract infection (e.g., pharyngitis, sinusitis, tonsillitis, laryngitis, tracheitis, epiglottitis, other disease of nasal cavity and sinuses, otitis) |
| 49302, 49312, 49322, 49392 | Asthma with acute exacerbation |
| **ICD-10 Codes** | **Condition** |
| A157 | Primary respiratory tuberculosis |
| A401 | Sepsis due to streptococcus, group B |
| A403 | Sepsis due to Streptococcus pneumoniae |
| A4101 | Sepsis due to Methicillin susceptible Staphylococcus aureus |
| A4102 | Sepsis due to Methicillin resistant Staphylococcus aureus |
| A411 | Sepsis due to other specified staphylococcus |
| A413 | Sepsis due to Hemophilus influenzae |
| A4151 | Sepsis due to Escherichia coli [E. coli] |
| A4153 | Sepsis due to Serratia |
| A4181 | Sepsis due to Enterococcus |
| A4189 | Other specified sepsis |
| A419 | Sepsis, unspecified organism |
| B250 | Cytomegaloviral pneumonitis |
| B348 | Other viral infections of unspecified site |
| B974 | Respiratory syncytial virus as the cause of diseases classified elsewhere |
| B9781 | Metapneumovirus as the cause of diseases classified elsewhere |
| C781 | Secondary malignant neoplasm of mediastinum |
| C8330 | Diffuse large B-cell lymphoma, unspecified site |
| C8350 | Lymphoblastic (diffuse) lymphoma, unspecified site |
| C8370 | Burkitt lymphoma, unspecified site |
| C8442 | Peripheral T-cell lymphoma, not classified, intrathoracic lymph nodes |
| D821 | Di George's syndrome |
| E440 | Moderate protein-calorie malnutrition |
| E7601 | Hurler's syndrome |
| E840 | Cystic fibrosis with pulmonary manifestations |
| E849 | Cystic fibrosis, unspecified |
| I330 | Acute and subacute infective endocarditis |
| I5021 | Acute systolic (congestive) heart failure |
| I5023 | Acute on chronic systolic (congestive) heart failure |
| I5030 | Unspecified diastolic (congestive) heart failure |
| I5033 | Acute on chronic diastolic (congestive) heart failure |
| I5041 | Systolic (congestive) and diastolic (congestive) heart failure |
| I509 | Heart failure, unspecified |
| J09X2 | Novel influenza A virus with other respiratory manifestations |
| J101 | Other identified influenza virus with other respiratory manifestations |
| J1089 | Influenza due to other identified influenza virus with other manifestations |
| J111 | Unidentified influenza virus with other respiratory manifestations |
| J1189 | Influenza due to unidentified influenza virus with other manifestations |
| J411 | Mucopurulent chronic bronchitis |
| J42 | Unspecified chronic bronchitis |
| J441 | Chronic obstructive pulmonary disease with (acute) exacerbation |
| J449 | Chronic obstructive pulmonary disease, unspecified |
| J4521 | Mild intermittent asthma with (acute) exacerbation |
| J4522 | Mild intermittent asthma with status asthmaticus |
| J4530 | Mild persistent asthma, uncomplicated |
| J4531 | Mild persistent asthma with (acute) exacerbation |
| J4532 | Mild persistent asthma with status asthmaticus |
| J4540 | Moderate persistent asthma, uncomplicated |
| J4541 | Moderate persistent asthma with (acute) exacerbation |
| J4542 | Moderate persistent asthma with status asthmaticus |
| J4550 | Severe persistent asthma, uncomplicated |
| J4551 | Severe persistent asthma with (acute) exacerbation |
| J4552 | Severe persistent asthma with status asthmaticus |
| J45901 | Unspecified asthma with (acute) exacerbation |
| J45902 | Unspecified asthma with status asthmaticus |
| J45909 | Unspecified asthma, uncomplicated |
| J45998 | Other asthma |
| J470 | Bronchiectasis with acute lower respiratory infection |
| J471 | Bronchiectasis with (acute) exacerbation |
| J698 | Pneumonitis due to inhalation of other solids and liquids |
| J80 | Acute respiratory distress syndrome |
| J811 | Chronic pulmonary edema |
| J8401 | Alveolar proteinosis |
| J8483 | Surfactant mutations of the lung |
| J8489 | Other specified interstitial pulmonary diseases |
| J849 | Interstitial pulmonary disease, unspecified |
| J851 | Abscess of lung with pneumonia |
| J860 | Pyothorax with fistula |
| J869 | Pyothorax without fistula |
| J90 | Pleural effusion, not elsewhere classified |
| J918 | Pleural effusion in other conditions classified elsewhere |
| J9311 | Primary spontaneous pneumothorax |
| J942 | Hemothorax |
| J95821 | Acute postprocedural respiratory failure |
| J9600 | Respiratory failure, unspecified whether with hypoxia or hypercapnia |
| J9601 | Acute respiratory failure with hypoxia |
| J9602 | Acute respiratory failure with hypercapnia |
| J9610 | Respiratory failure, unspecified whether with hypoxia or hypercapnia |
| J9620 | Respiratory failure, unspecified whether with hypoxia or hypercapnia |
| J9621 | Acute and chronic respiratory failure with hypoxia |
| J9622 | Acute and chronic respiratory failure with hypercapnia |
| J9690 | unspecified, unspecified whether with hypoxia or hypercapnia |
| J9691 | Respiratory failure, unspecified with hypoxia |
| J9692 | Respiratory failure, unspecified with hypercapnia |
| J9801 | Acute bronchospasm |
| J9811 | Atelectasis |
| J984 | Other disorders of lung |
| J988 | Other specified respiratory disorders |
| J989 | Respiratory disorder, unspecified |
| P0703 | Extremely low birth weight newborn, 750-999 grams |
| P0714 | Other low birth weight newborn, 1000-1249 grams |
| P0722 | Immaturity of newborn, gestational age 23 completed weeks |
| P0723 | Immaturity of newborn, gestational age 24 completed weeks |
| P220 | Respiratory distress syndrome of newborn |
| P228 | Other respiratory distress of newborn |
| P229 | Respiratory distress of newborn, unspecified |
| P2401 | Meconium aspiration with respiratory symptoms |
| P2481 | Other neonatal aspiration with respiratory symptoms |
| P251 | Pneumothorax originating in the perinatal period |
| P271 | Onchopulmonary dysplasia originating in the perinatal period |
| P285 | Respiratory failure of newborn |
| P2889 | Other specified respiratory conditions of newborn |
| P289 | Respiratory condition of newborn, unspecified |
| P290 | Neonatal cardiac failure |
| P293 | Persistent fetal circulation |
| P360 | Sepsis of newborn due to streptococcus, group B |
| P3619 | Sepsis of newborn due to other streptococci |
| P362 | Sepsis of newborn due to Staphylococcus aureus |
| P3630 | Sepsis of newborn due to unspecified staphylococci |
| P369 | Bacterial sepsis of newborn, unspecified |
| P760 | Meconium plug syndrome |
| P9160 | Hypoxic ischemic encephalopathy [HIE], unspecified |
| P9683 | Meconium staining |
| Q210 | Ventricular septal defect |
| Q234 | Hypoplastic left heart syndrome |
| Q250 | Patent ductus arteriosus |
| Q330 | Congenital cystic lung |
| Q332 | Sequestration of lung |
| Q336 | Congenital hypoplasia and dysplasia of lung |
| Q339 | Congenital malformation of lung, unspecified |
| Q341 | Congenital cyst of mediastinum |
| Q790 | Congenital diaphragmatic hernia |
| R05 | Cough |
| R0600 | Dyspnea, unspecified |
| R062 | Wheezing |
| R0902 | Hypoxemia |
| R092 | Respiratory arrest |
| R571 | Hypovolemic shock |
| R6520 | Severe sepsis without septic shock |
| R7881 | Bacteremia |
| T17508A | Unspecified foreign body in bronchus causing other injury, initial encounter |
| T17590A | Other foreign object in bronchus causing asphyxiation, initial encounter |
| T17890A | Other foreign object in other parts of respiratory tract causing asphyxiation, initial encounter |
| T17920A | Food in respiratory tract, part unspecified causing asphyxiation, initial encounter |
| T751XXA | Unspecified effects of drowning and nonfatal submersion, initial encounter |
| A360, A361, A362, A3689, J00-J06, J32, J340, J341, J3481, J3489, J349, J35, H60, H6190, H6191, H6192, H6193, H624, H628X1, H628X2, H628X3, H628X9, H65-H67 | Upper respiratory tract infection (e.g., pharyngitis, sinusitis, tonsillitis, laryngitis, tracheitis, epiglottitis, other disease of nasal cavity and sinuses, otitis) |
| J4521, J4531, J4541, J4551, J441, J45901 | Asthma with acute exacerbation |
